# The PHD1 finger of KDM5B recognizes unmodified H3K4 during the demethylation of histone H3K4me2/3 by KDM5B

**DOI:** 10.1007/s13238-014-0078-4

**Published:** 2014-06-22

**Authors:** Yan Zhang, Huirong Yang, Xue Guo, Naiyan Rong, Yujiao Song, Youwei Xu, Wenxian Lan, Xu Zhang, Maili Liu, Yanhui Xu, Chunyang Cao

**Affiliations:** 1 State Key Laboratory of Bio-organic and Natural Product Chemistry, Shanghai Institute of Organic Chemistry, Chinese Academy of Sciences, Shanghai, 200032 China; 2 Institutes of Biomedical Sciences, Fudan University, 130 Dong-An Road, Shanghai, 200032 China; 3 State Key Laboratory of Magnetic Resonance and Atomic and Molecular Physics, Wuhan Institute of Physics and Mathematics, Chinese Academy of Sciences, Wuhan, 430071 China

**Keywords:** KDM5B, PHD1, H3K4me0, demethylase, repression, structure

## Abstract

KDM5B is a histone H3K4me2/3 demethylase. The PHD1 domain of KDM5B is critical for demethylation, but the mechanism underlying the action of this domain is unclear. In this paper, we observed that PHD1_KDM5B_ interacts with unmethylated H3K4me0. Our NMR structure of PHD1_KDM5B_ in complex with H3K4me0 revealed that the binding mode is slightly different from that of other reported PHD fingers. The disruption of this interaction by double mutations on the residues in the interface (L325A/D328A) decreases the H3K4me2/3 demethylation activity of KDM5B in cells by approximately 50% and increases the transcriptional repression of tumor suppressor genes by approximately twofold. These findings imply that PHD1_KDM5B_ may help maintain KDM5B at target genes to mediate the demethylation activities of KDM5B.

**Electronic supplementary material:**

The online version of this article (doi:10.1007/s13238-014-0078-4) contains supplementary material, which is available to authorized users.

## Introduction

Covalent histone modifications, notably methylation, are reversible posttranslational modifications that play key roles in chromatin structure, gene transcription and the epigenetic state of the cell (Martin and Zhang, 2005; Mosammaparast and Shi, 2010). Six lysine residues, including histones H3 (i.e., H3K4, H3K9, H3K27, H3K36 and H3K79) and H4 (i.e., H4K20), can be mono-, di-, or trimethylated, and each modification is found in a stereotypical pattern with respect to the coding region of a gene and correlates with a different transcriptional outcome (Zhang and Reinberg, 2001; Margueron et al., 2005). As a general rule, the methylation of H3K9, H3K27 and H4K20 is associated with transcriptional repression, whereas the methylation of H3K4, H3K36 and H3K79 is related to transcriptional activation (Mosammaparast and Shi, 2010; Zhang and Reinberg, 2001). The methylation of H3K4 is a key regulator for RNA polymerase binding to an active gene (Sims et al., 2003) and of transcription factor binding within promoter elements (Szutorisz et al., 2005). One of the important aspects of H3K4 methylation is how this epigenetic mark is removed, thereby reducing the localization of RNA polymerase to the specific genes. The loss of H3K4 methylation appears to be a key step of differentiation (Mikkelsen et al., 2007). To date, two distinct classes of histone demethylases have been characterized. The first class includes two members, represented by LSD1 (lysine-specific demethylase 1, also known as KDM1A, which demethylates H3K4me1/2) and LSD2 (also called KDM1B or AOF1, which demethylates H3K4me2). Both members of this class use FAD as a cofactor through an amine-oxidation reaction to remove the lysine methyl group of H3K4me1/2 (Shi et al., 2004; Ciccone et al., 2009). The other class contains a JmjC (i.e., Jumonji C) domain to catalyze histone lysine demethylation assisted by two cofactors: Fe^2+^ ion and α-ketoglutarate (α-KG) (Chen et al., 2006; Cloos et al., 2006; Tsukada et al., 2006; Whetstine et al., 2006, Shi and Whetstine, 2007). Based on the sequence homology in the JmjC domain and the overall architecture of the associated motifs, JmjC domain-containing proteins have been classified into seven groups: JHDM1, PHF2/8, JARID, JHDM3/JMJD2, UTX/UTY, JHDM2 and JmjC domain only (Chen et al., 2006). Structural investigations of the members of this class, including JHDM1A, JHDM1D, JMJD2A and PHF8, have already been performed for their apo forms or in complex with the H3 peptide and α-KG substrates (Chen et al., 2006; Chen et al., 2007; Couture et al., 2007; Ng et al., 2007; Horton et al., 2010; Yang et al., 2010). However, no structure has been published for the members of the JARID1 sub-group that can specifically remove methyl groups of di- or tri-methylated H3K4.

**Figure 1:**
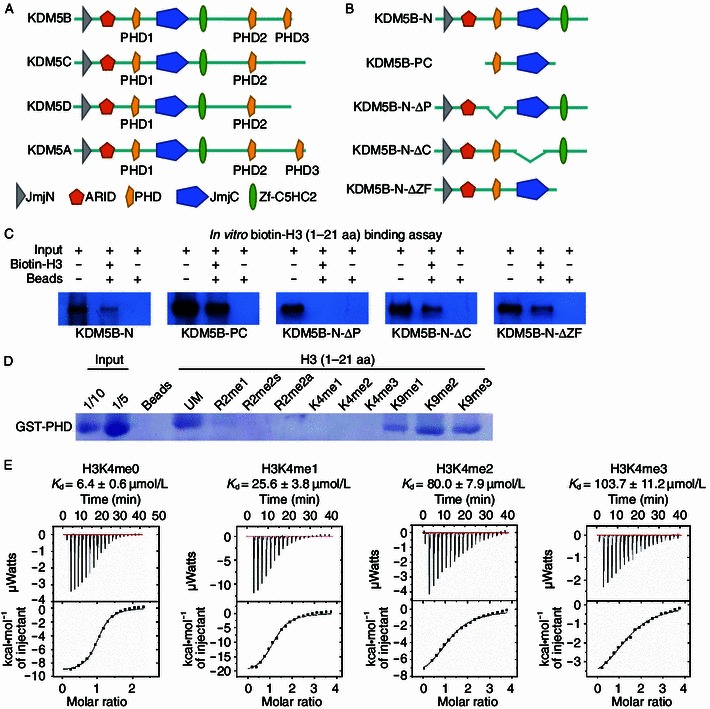
**PHD1**_**KDM5B**_**specifically binds to the tail of H3K4me0**. (A) Mammalian KDM5 family members are highly similar in domain architecture and contain JmjN, ARID, Jmjc, zf-C5HC2 and PHD domains. (B) KDM5B variants described in the text. (C) *In vitro* binding assays for analysis of the binding of recombinant KDM5B variants to the unmodified histone H3K4 N-terminal tail. (D) PHD1_KDM5B_ is sufficient for H3 tail binding. (E) The binding affinities of PHD1_KDM5B_ to unmodified, mono-methylated, di-methylated or tri-methylated H3K4 peptides (residues 1–10) were measured through an ITC assay. The *K*_D_ values are the means (± s.d.) of at least three experiments using varied peptide and protein concentrations

The members of the JARID1 subgroup are highly conserved from yeast to humans and contain a similar motif architecture, including JmjN, ARID (i.e., AT-rich interactive domain), JmjC, Zf-C_5_HC_2_ (i.e., zinc finger) and two or three PHD domains (denoted PHD1, PHD2 and PHD3 from the N-terminus to the C-terminus). A total of four members are found in mammals (Fig. 1A): JARID1A (also called RBP2 or KDM5A), JARID1B (also named PLU-1 or KDM5B), JARID1C (i.e., SMCX or KDM5C) and JARID1D (also known as SMCY orKDM5D). These members have been identified to be H3K4me2/3 demethylases (Christensen et al., 2007; Iwase et al., 2007; Klose et al., 2007; Lee et al., 2007; Tahiliani et al., 2007; Yamane et al., 2007). All of these proteins are key transcriptional co-repressors because they can remove the transcription-activating marker H3K4me3. KDM5B is involved in transcriptional repression and breast cancer cell proliferation (Yamane et al., 2007); thus, mechanistic studies on KDM5B demethylation are necessary and useful to understand the development of breast cancer. Notably, the deletion of the N-terminal PHD1 finger (i.e., PHD1_KDM5B_) of KDM5B results in the loss of enzymatic demethylase activity, implying that PHD1_KDM5B_ is involved in H3K4me2/3 demethylation (Yamane et al., 2007). This observation is consistent with the fact that the N-terminal PHD1 finger of Lid (i.e., PHD1_Lid_), a homologue of KDM5B in *Drosophila*, is also required for the demethylase activity of H3K4me3, whereas the PHD2 and PHD3 of Lid are not (Li et al., 2010). However, the detailed mechanism underlying the function of PHD1_KDM5B_ in the demethylation process remains unclear.

Recently, the NAD^+^-dependent PARylation on KDM5B by poly (ADP-ribose) polymerase (PARP-1) was reported to be able to regulate chromatin structure and transcription through a KDM5B-dependent pathway (Krishnakumar and Kraus, 2010). The demethylation inhibited by PARylation on KDM5B was confirmed by an *in vitro* H3 binding assay. Previously, PHD1_Lid_ was reported to bind with unmodified H3K4me0 (Li et al., 2010). These observations indicate that KDM5B may bind to the H3 peptide, most likely to unmodified H3K4. Thus, in this study, we first tested the interactions of the unmodified H3K4 peptide with full-length KDM5B or its truncated variants through a biotin-labeled peptide binding assay. By sequentially depleting different regions in KDM5B-N, we found that PHD1 in KDM5B (i.e., PHD1_KDM5B_) can specifically bind to the unmodified histone H3. To probe the structural basis for this interaction, we determined a solution structure of PHD1_KDM5B_ in complex with the unmodified H3K4 peptide. Through structural and biochemical data, we provide insights into the function of PHD1_KDM5B_ in KDM5B-regulated demethylation and tumor-suppressor gene transcription.

## Results

### PHD1_KDM5B_ specifically interacts with the unmodified H3K4me0 peptide

To investigate the function of PHD1_KDM5B_ in the demethylation of H3K4me2/3 by KDM5B (as shown in Fig. 1B), the binding affinities of five recombinant KDM5B variants, namely KDM5B-N (N-terminal KDM5B), KDM5B-PC (only containing PHD1 and JmjC domains in KDM5B-N), KDM5B-N-△P (without the PHD1 domain in KDM5B-N), KDM5B-N-△C (without the JmjC domain in KDM5B), and KDM5B-N-△ZF (without the Zf-C_5_HC_2_ domain in KDM5B-N), were tested with a biotin-labeled unmodified H3 peptide (Fig. 1C). The results indicate that KDM5B-N interacts with the unmodified H3 peptide. Similar to the KDM5B-N variant, the KDM5B-PC, KDM5B-N-△C and KDM5B-N-△ZF variants bind to the unmodified H3K4 peptide, implying that the JmjC domain, the Zf-C_5_HC_2_ domain, and the JmjN-ARID domain are not involved in the interaction with the unmodified H3K4 peptide. However, the deletion of the N-terminal PHD1_KDM5B_ domain in KDM5B-N (i.e., KDM5B-N-△P) significantly impaired the interaction between KDM5B-N and the H3K4me0 peptide. This result suggested that PHD1_KDM5B_ primarily contributes to the binding of KDM5B to the H3K4me0 peptide. The glutathione S-transferase (GST) tag-fused PHD1_KDM5B_ (306–360 aa, similarly hereinafter) protein binds to the unmodified H3 tail (Fig. 1D), further supporting the function of PHD1_KDM5B_ in the specific binding to the unmodified H3. The methylation of H3R2 and H3K4 abolishes or weakens the binding affinity, suggesting that these two amino acids may be involved in the binding. In addition, the methylation of H3K9 does not notably inhibit this binding, which indicates that H3K9 does not participate in the binding. Moreover, an ITC analysis obtained the dissociation constants (*K*_D_) of 6.4 ± 0.6 μmol/L for PHD1_KDM5B_ interacting with the unmodified H3K4 peptide, 25.6 ± 3.8 μmol/L for PHD1_KDM5B_ interacting with mono-methylated H3K4me1, 80.0 ± 7.9 μmol/L for PHD1_KDM5B_ interacting with dimethylated H3K4me2 and 103.7 ± 11.2 μmol/L for PHD1_KDM5B_ interacting with tri-methylated H3K4me3 (Fig. 1E). These *K*_D_s revealed that H3K4 is highly involved in the binding and that the methylation of H3K4 inhibits this binding.

### Solution structure of PHD1_KDM5B_ in complex with H3K4me0

**Figure 2:**
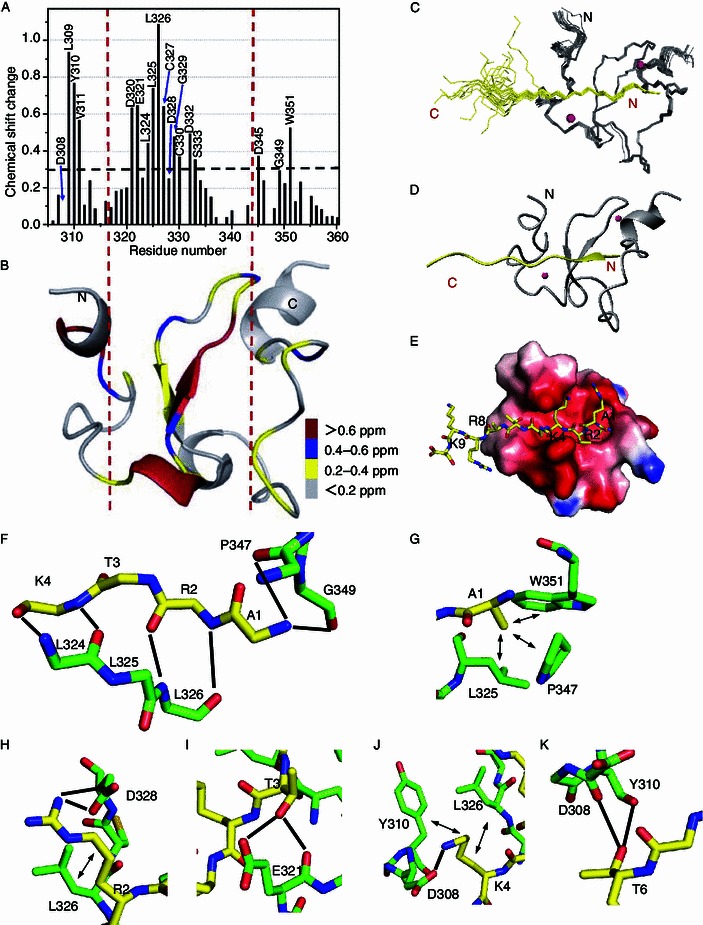
**Structure of PHD1**_**KDM5B**_**with the unmodified H3 tail (1–10 aa)**. (A) An NMR titration assay was used to map the binding sites of the H3K4me0 peptide on PHD1_KDM5B_. The chemical shift of the PHD1_KDM5B_ backbone atoms amide ^1^H and ^15^N after H3K4me0 peptide binding was calculated using the following equation: $\Delta \delta_{\text{av}}={\left\{0.5\times \left[\Delta \delta ({\text{NH)}}^{2}+0.2\;\times \;\Delta \delta {\left({}^{15}\text{N}\right)}^{2}\right]\right\}}^{1/2}$. (B) Ribbon representation of free PHD1_KDM5B_. To indicate the binding sites of the H3K4me0 peptide on PHD1_KDM5B_, the residues with different chemical shifts are marked in different colors. (C) Backbone atoms (N, C_α_ and C′) of the 20 superposed NMR structures of PHD1_KDM5B_ (grey) in complex with the H3K4me0 peptide (yellow). (D) The ribbon representation of the structure of the complex highlights the secondary structural elements (protein, grey; peptide, yellow). The pink spheres represent zinc atoms. For clarity, only the representation of the zinc atoms in the lowest energy structure is shown in the ensemble. (E) Electrostatic potential surface representation of the PHD1_KDM5B_ interaction with the H3K4me0 peptide (yellow). The residues in the H3 peptide are labeled. The orientations of the protein in figures (B) to (E) are identical. (F) The hydrogen-bond interactions between the backbone atoms of PHD1_KDM5B_ and the H3K4me0 peptide. (G–K) Key protein-peptide side-chain interactions between A1, R2, T3, K4 and T6 in the H3 peptide and residues in PHD1_KDM5B_. The carbon atoms in the peptide and protein residues are shown in yellow and green, respectively. The nonpolar non-bonded interacting atoms are labeled with ⟷

To understand the interaction of PHD1_KDM5B_ with unmodified H3K4, we initially attempted to crystallize PHD1_KDM5B_ in its free form and in complex with the H3K4me0 peptide. However, we only obtained the X-ray structure of the free PHD1_KDM5B_ with a high resolution of 1.65 Å (Fig. S1) (Guo et al., 2011). Thus, we determined the solution structures of free PHD1_KDM5B_ and of PHD1_KDM5B_ in complex with the H3K4 peptide (1–10 aa) using multidimensional heteronuclear NMR spectroscopy (Fig. 2). To probe whether the electronic properties of histidines in solution are similar to those in the crystal state (two histidines, H335 and H344, are in the amino acid sequence of PHD1_KDM5B_; the H335 residue was suggested to ligate with a zinc ion in the X-ray crystal structure of free PHD1_KDM5B_) before structural determination, we analyzed the ^1^H-^15^N LR-HSQC spectra of PHD1_KDM5B_ in the free and bound states (Fig. S2). This LR-HSQC experiment correlates the carbon-bound protons of the histidine rings with the imidazole nitrogen atoms and can unambiguously establish the tautomeric and protonation states of histidines in proteins (Pelton et al., 1993; Drohat et al., 1999). The characteristic upside-down L-shaped patterns of the peaks in the LR-HSQC spectra for the H335 and H344 residues and their well-separated ^15^N chemical shifts indicated that these two histidines are in the Nε2-H tautomeric form. Additionally, both histidines are in their neutral form under the NMR experimental conditions. The ^15^Nδ1 chemical shift of H335 is more deshielded than that of H344 in PHD1_KDM5B_, revealing the strong chelation of H335 to an electropositive Zn^2+^. This observation is identical to that observed in the X-ray structure of the free PHD1_KDM5B_.

The NMR solution structure of free PHD1_KDM5B_ (Fig. 2B) was then determined using the program XPLOR-NIH (Kuszewski and Clore, 2000) with 670 NOEs, 10 hydrogen bonds and 106 dihedral angle constraints that were meaningful and acceptable (Table 1). The resulting structure was similar to that obtained by X-ray techniques with a backbone-atom RMSD of 0.48 Å, which was determined by superimposing the backbone C_α_ atoms in the secondary structural region (Fig. S1). Thus, in this paper, we only use the NMR structure of the free PHD1_KDM5B_ for comparison with that of the complex. In contrast, the solution structure of PHD1_KDM5B_ in complex with an unmodified H3K4me0 peptide (1–10 aa) was obtained through 1263 NOEs (including 150 observed intermolecular NOEs), 17 hydrogen bonds and 106 dihedral angle constraints. The PHD1_KDM5B_ structures in both free and bound states adopt a ‘cross-braced’ topology of zinc ion coordinated residues that was identical to that of all structurally characterized PHD fingers. These states are well defined by NMR data; the RMSD of the free PHD1_KDM5B_ and the PHD1_KDM5B_ in complex with the H3 peptide were 1.18 ± 0.17 Å and 0.63 ± 0.14 Å for the heavy atoms in the secondary structural regions of the 20 lowest energy structures, respectively. By superimposing the backbone C_α_ atoms in the secondary structural region, the free and bound solution PHD1_KDM5B_ structures have a backbone-atom RMSD value of 1.13 Å (Fig. S1). This RMSD indicates that the H3K4me0 peptide binding does not induce major conformational changes to the backbone of PHD1_KDM5B_.

**Table 1:** NMR structural statistics for free PHD1_KDM5B_ and for the complex of PHD1_KDM5B_ with H3K4me0 peptide

NMR distance and dihedral constraints	PHD1-H3K4me0	PHD1-free
Distance restraints from NOEs		
Intra-molecualr		
Total	1263	670
Intraresidue (|i − j| = 0)	445	232
Sequential (|i − j| = 1)	214	214
Medium range (1 < |i − j| < 5)	218	118
Long range (|i − j| > 5)	236	106
Inter-molecular	150	-
H-bonds	17	10
Dihedral angle constraints	106	106
φ	53	53
ψ	53	53
Structure statistics		
Rms deviations versus the mean structure (Å)		
All backbone atoms	0.74 ± 0.19	1.13 ± 0.24
All heavy atoms	1.34 ± 0.20	1.72 ± 0.27
Backbone atoms (secondary structure)	0.16 ± 0.04	0.71 ± 0.19
Heavy atoms (secondary structure)	0.63 ± 0.14	1.18 ± 0.17
Rms deviations from experimental restraints		
NOE distance (Å)	0.035 ± 0.011	0.096 ± 0.015
Dihedral angles (deg.)	0.79 ± 0.20	1.6 ± 0.16
Rms deviations from idealized geometry		
Bonds (Å)	0.0034 ± 0.00026	0.0020 ± 0.00014
Angles (deg.)	0.91 ± 0.073	0.41 ± 0.073
Impropers (deg.)	0.47 ± 0.032	0.47 ± 0.053
Structure analysis		
Residues in most favored regions	82.5	84.9
Residues in additionally allowed regions	17.3	9.8
Residues in generously allowed regions	0.3	5.3
Residues in disallowed regions	0	0

In the structure of the PHD1_KDM5B_-H3K4me0 complex, the H3 peptide binds to the surface of the PHD1_KDM5B_ as an anti-parallel β-sheet (residues 2–3 aa). This H3 β-sheet is shorter in this complex than in the complex of the PHD of human autoimmune regulator, (PHD_AIRE_) with unmethylated H3K4 (PDB code: 2KFT)(Chakravarty et al., 2009) and in the complex of the PHD finger of the BHC80 protein in the LSD1 co-repressor (PHD_BHC80_) with unmethylated H3K4 (PDB code: 2PUY)(Lan et al., 2007) (Fig. 2D). In the current structure of the complex, the backbone atoms of residues H3R2 and H3K4 in the unmethylated H3 peptide form hydrogen bonds with the backbone carbonyl oxygen and nitrogen atoms of L324 and L326 in PHD1_KDM5B_ (Fig. 2F), respectively. The cognate PHD1_KDM5B_ only contacts the first six residues of the H3 peptide, whereas H3K9 is completely exposed to solvent by extending its side chain away from the complex (Fig. 2E). This finding supports the concept that the methylation of H3K9 has no effect on the binding. The conformation of the side chain of H3R8 is also flexible. In the 20 final NMR structures for the complex, H3R8 does not contact the protein. This coincides with the fact that no intermolecular NOEs were observed between the H3R8 side chain and the protein.

### Analysis of the interaction between PHD1_KDM5B_ and the unmodified H3K4me0 peptide

According to the structural information provided by the structure of the complex of PHD1_KDM5B_ with the unmodified H3K4 peptide, the H3K4me0 specificity of PHD1_KDM5B_ is determined through the recognition of the residues in the H3 amino terminus, including H3A1, H3R2, H3T3, H3K4 and H3T6 (Fig. 2F–K). The H3A1 methyl group is anchored by intermolecular hydrogen bonds with the backbone carbonyl oxygen atoms of residues P347 and G349 (Fig. 2F, in which the H3A1 backbone nitrogen supplies two hydrogen bonds) and by nonpolar hydrophobic interactions between the H3A1 methyl group and the side chains of L325, P347 and W351 in PHD1_KDM5B_ (Fig. 2G). These side chains are similar to those observed in the PHD_AIRE_-H3K4me0 complex and other complexes (Chakravarty et al., 2009; Lan et al., 2007, Li et al., 2006, Pena et al., 2006, Hu et al., 2011, Wang et al., 2011). These observations support our site-directed mutation studies for both the PHD1_KDM5B_ protein and the H3K4me0 peptide (Table S1). On the one hand, both the W351A and L325A mutants of PHD1_KDM5B_ have non-detectable binding affinities with the H3K4me0 peptide. On the other hand, removing the methyl group from H3A1 by changing alanine to glycine results in an approximately 50-fold decrease in the binding affinity of the unmodified H3 peptide to PHD1_KDM5B_ (*K*_D_^H3K4-PHD1^ = 6.4 ± 0.6 μmol/L and *K*_D_^H3 A1G-PHD1^ = 304.9 ± 11.7 μmol/L). In the ^1^H-^15^N HSQC spectra, compared with that of free PHD1_KDM5B_, the binding to wild-type H3A1 (i.e., H3K4me0) produces a large shift in most of the cross-peaks of PHD1_KDM5B_, whereas the binding of the H3G1 variant does not induce this shift. This result indicates the important roles of the H3A1 methyl group in the interaction. One of the positively charged side-chain nitrogen atoms (Nη) of H3R2 forms a salt bridge with one of the negatively charged oxygen atoms in the side-chain of D328 (Fig. 2H). D328 mutations in PHD1_KDM5B_ (the D328A mutant) or R2 mutations in the H3K4me0 peptide (H3 R2A or R2E mutants) remove the negative or positive charges in their side chains; therefore, the binding affinities were significantly reduced (*K*_D_^H3A1-PHD1 D328A^ = 182.1 ± 21.9 μmol/L, the binding affinity decreased by 30-fold; *K*_D_^H3 R2A-PHD1^ = 370.4 ± 52.1 μmol/L, the binding affinity decreased by 60-fold; and the *K*_D_ value for the interaction of PHD1_KDM5B_ to the H3 R2E mutant was too small to detect) (Table S1) compared with that of the wild-type PHD1_KDM5B_ or the unmodified H3K4 peptide. These results were consistent with the observations mentioned for the above-described structures. In addition, the distance between the Cβ atom of L326 and the Cβ atom of H3R2 is 4.0 Å, indicating a hydrophobic interaction between the side chains of L326 and H3R2 (Fig. 2H).

The side-chain -OH group of H3T3 forms two additional hydrogen bonds with one of the side-chain carbonyl oxygen atoms and the backbone oxygen of E321 (Fig. 2I), further stabilizing the interaction between the H3 peptide and the PHD1_KDM5B_ protein. Removing hydrogen bonds by adding mutations from E321 to A321 in PHD1_KDM5B_ or from T3 to V3 in the H3 peptide lowers the binding affinities of the H3K4me0 peptide to PHD1_KDM5B_ by approximately tenfold (*K*_D_^H3K4-PHD1 E321A^ = 75.8 ± 5.7 μmol/L, *K*_D_^H3K4 T3V-PHD1^ = 57.1 ± 2.2 μmol/L). The distances between the H3T6-OH group and the backbone carboxyl oxygen of D308 and Y310 are less than 4 Å (Fig. 2K), indicating that weak hydrogen bonds might form between these atoms. The presence of these weak hydrogen bonds is supported by the measured *K*_D_ values of the mutants (*K*_D_^H3 T6V-PHD1^ = 61.3 ± 1.6 μmol/L, the binding affinity decreased by approximately tenfold compared with that of the wild-type protein, which exhibits a *K*_D_^H3K4-PHD1^ of 6.4 ± 0.6 μmol/L).

Moreover, the side-chain NH_3_^+^ group of H3K4 forms a rigid hydrogen bond with the side-chain carbonyl oxygen of D308 (Fig. 2J), which supports the results of the analysis of the structure of the complex and of the mutation studies (*K*_D_^H3 K4E-PHD1^ is non-detectable, whereas *K*_D_^H3K4-PHD1 D308A^ was measured as 41.7 ± 2.2 μmol/L). In addition to interacting with D308, the side chain of H3K4 also displays weak hydrophobic interactions with the aromatic ring of Y310 and the methyl groups of L326 (Fig. 2J). The distances between the Cγ or Cδ atom of the H3K4 side chain and the aromatic Cγ atom of Y310 or the methyl group of L326 in PHD1_KDM5B_ are approximately 4.5 Å. The mutation of Y310 to F310 does not change the binding affinities of PHD1_KDM5B_ to the unmodified H3 peptide (*K*_D_^H3K4-PHD1^ = 6.4 ± 0.6 μmol/L and *K*_D_^H3K4-PHD1 Y310F^ = 4.3 ± 0.2 μmol/L), suggesting that the-OH group in the side chain of residue Y310 may not be involved in the interaction. When Y310 is replaced by A310, the binding affinity between PHD1_KDM5B_ and the H3 peptide decreased by approximately fivefold (*K*_D_^H3K4-PHD1 Y310A^ = 28.1 ± 1.3 μmol/L) compared with the binding affinity of the wild-type PHD1_KDM5B_ to the H3 peptide. The PHD1_KDM5B_ L326A mutant has an approximately two-fold weaker binding affinity (*K*_D_^H3K4-PHD1 L326A^ = 14.1 ± 0.8 μmol/L) to the unmodified H3 peptide than the wild-type. These observations imply that the hydrophobic interactions between the side chains of L325 and Y310 in PHD1_KDM5B_ and H3K4 contribute less to the binding than the hydrogen bond between H3K4 and D308 in PHD1_KDM5B_. The methylation of H3K4 may weaken these hydrophobic interactions because minimal space exists among residues L325 and Y310 in PHD1_KDM5B_ and H3K4. This may explain the reduction in the binding affinities of PHD1_KDM5B_ to H3K4me1 (by ~4-fold), H3K4me2 (by >10-fold) and H3K4me3 (by >15-fold) compared with the H3K4me0 peptide. Therefore, molecular recognition of the unmodified lysine 4 primarily occurs through hydrogen bonding to the unmodified epsilon amino group and steric elusion of the methyl groups on H3K4me3/2.

**Figure 3:**
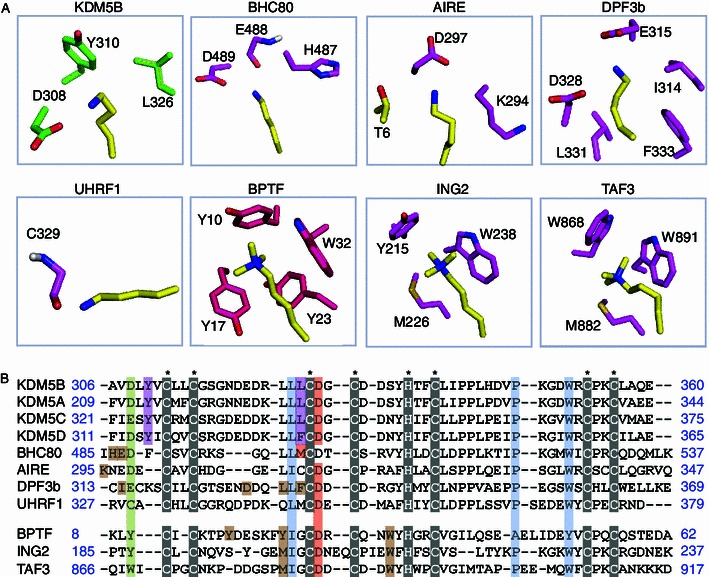
**Binding mode of PHD1**_**KDM5B**_**to the H3K4me0 peptide**. (A) The recognition modes of H3K4me0 by PHD1_KDM5B_, PHD_BHC80_, PHD_ARIE_, PHD2_DPF3b_ and PHD_UHRF1_ are displayed. For comparison, the recognition of H3K4me3 by PHD_BPTF_, PHD_ING2_ and PHD_TAF3_ are also shown. Either H3K4me0 or H3K4me3 is depicted in yellow. (B) Sequence alignment of the PHD fingers (binding to H3K4me0) of KDM5 family members (BHC80, AIRE, DPF3b and UHRF1) and of the PHD fingers (binding methylated H3K4) of BPTF, ING2 and TAF3. The zinc-binding residues, H3A1-binding residues and H3R2-binding residues are highlighted in grey, blue and red, respectively. The H3K4-binding residues are highlighted in purple (in the KDM5 family), green (in all of the PHD fingers) and brown (in all of the PHD fingers except those of the KDM5 family)

In addition, residues D308, Y310 and L326 are conserved among the members in the KDM5 PHD1 family (Fig. 3B) with the exception of residues E323 in KDM5C and F331 in KDM5D. Therefore, our structure may reveal that the PHD1 domain in other members of the KDM5 family functions as a specific reader of unmodified H3K4, although structural models of the PHD1 KDM5A, KDM5C and KDM5D in complex with unmodified H3K4 are not available.

### Recognition of H3K4me0 by PHD1_KDM5B_ affects KDM5B demethylase activity

The binding sites of unmodified H3K4 on PHD1_KDM5B_ were first determined by an NMR titration binding assay (Fig. 2A and 2B). The majority of residues with chemical shifts larger than 0.3 ppm are involved in the interaction; this result is consistent with the results from the ITC assay (Table S1) and with the structural information of the complex. In the solution structure of PHD1_KDM5B_ in complex with H3K4, residues D308, L325, D328 and W351 are involved in the interaction between PHD1_KDM5B_ and H3K4 through hydrogen bonds, hydrophobic interactions, or salt bridges (Fig. 2). The single-site mutation of D308, L325, D328 or W351 to alanine disrupts the interaction between PHD1_KDM5B_ and the H3K4me0 peptide. Based on these structural and biochemical analyses, we then designed three full-length KDM5B mutants (D308A, L325A/D328A and W351A) and tested whether the *in vivo* H3K4me2/3 demethylation by KDM5B is affected by disrupting the interaction between PHD1_KDM5B_ and H3K4me0 through an immunofluorescence staining assay.

**Figure 4:**
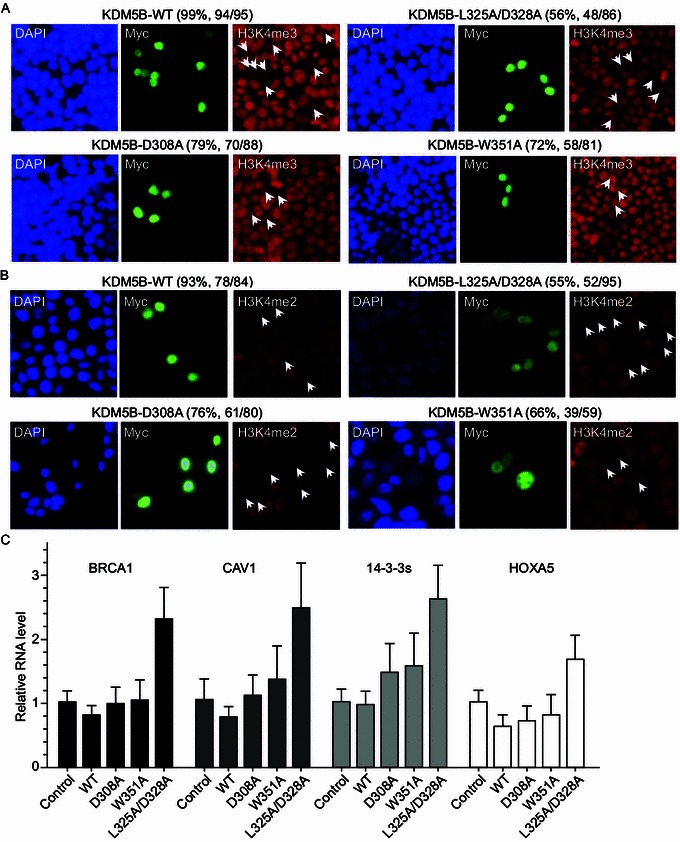
**PHD1**_**KDM5B**_**binding to H3K4me0 is important for KDM5B demethylase activity and KDM5B-mediated repression**. Various mutants of KDM5B-myc proteins were expressed in 293T cells, and the histone modification levels were analyzed by immunofluorescent staining with (A) H3K4me3 and (B) H3K4me2 specific antibodies. The arrows indicate KDM5B-transfected cells. (C) KDM5B-mediated repression was affected by the disruption of the interaction between PHD1_KDM5B_ and unmodified H3K4. The expression levels of BRCA1, CAV1, 14-3-3s and HOXA5 were analyzed in cells expressing wild-type and mutant KDM5B by real-time RT-PCR

Compared with the wild-type (WT) KDM5B (99% of H3K4me3 was demethylated), the D308A mutant exhibited 79% demethylase activity on H3K4me3 (decreased by approximately 20%) (Fig. 4A). The mutants that completely lose their ability to binding to the unmodified H3K4 exhibited decreases in their demethylase activities to 72% (W351A) and 56% (L325A/D328A). For H3K4me2 demethylation (Fig. 4B), the wild-type KDM5B displays 93% demethylase activity, whereas the D308A, W351A and L325A/D328A mutants demonstrate 76%, 66% and 55% demethylase activities, respectively. In both cases, the L325A/D328A mutant has a higher effect on the demethylase activities of KDM5B. These data indicate that the KDM5B demethylase activity was partially affected by (but not dependent on) the interaction between the N-terminal PHD1 finger and the unmodified N-terminal H3K4 tail.

### Recognition H3K4me0 by PHD1_KDM5B_ affects repression of tumor suppressor genes

KDM5B has been reported to function as a transcriptional repressor, and the knockdown of KDM5B increases the expression level of several tumor suppressor genes, including 14-3-3s, BRCA1, CAV1 and HOXA5 (Tan et al., 2003; Yamane et al., 2007). To illustrate the functional importance of the binding between PHD1_KDM5B_ and the unmodified H3K4, we tested whether the mutants could affect the expression of tumor suppressor genes compared with wild-type KDM5B. As shown in Fig. 4C, the overexpression of WT KDM5B decreased the expression of tumor suppressor genes. Compared with WT KDM5B, the overexpression of the D308A, W351A and L325A/D328A mutants up-regulated the expression of the four genes to a lesser but still significant extent. Among the mutants, the L325A/D328A mutant increased the gene expression by more than twofold. Therefore, the gene expression results agree with the binding affinities of PHD1_KDM5B_ and its mutants with the H3 peptide. The overexpression of the mutants is expected to produce fewer unmodified H3K4, thereby increasing the gene repression level. In combination, these findings support the model in which PHD1_KDM5B_ specifically binds to the unmodified histone H3. Additionally, the H3 histone remains unmodified. This process is correlated with KDM5B demethylase activity and KDM5B-mediated gene repression.

## Discussion

### Comparison between PHD1_KDM5B_ and other PHD fingers

The reported structure of PHD_BHC80_ in complex with unmodified H3K4 (PDB code: 2PUY) demonstrated that H3K4 residue forms hydrogen bonds with the side chains of D489 and E488 (Fig. 3A) (Lan et al., 2007). Moreover, the Cβ atom of H487 restricts the interaction of PHD_BHC80_ with methylated H3K4, which means that the H3K4me1 and H3K4me2 peptides cannot bind to PHD_BHC80_ (Lan et al., 2007). However, in the PHD1_KDM5B_-H3K4me0 complex, H3K4me0 recognition is enhanced by hydrophobic interactions of the side-chains of H3K4 with the aromatic ring of Y310 and the methyl group of L326. Therefore, the binding mode of PHD1_KDM5B_-H3K4me0 differs slightly from that of PHD_BHC80_. Recognition of H3K4me0 in PHD1_KDM5B_, PHD_BHC80_, PHD_ARIE_ (Chakravarty et al., 2009), the PHD finger in UHRF1 (i.e., PHD_UHRF1_, PDB code: 2LGG) (Hu et al., 2011; Wang et al., 2011; Lallous et al., 2011; Rajakumara et al., 2011) and the PHD2 finger in DPF3b (i.e., PHD2_DPF3b_, PDB code 2KWK) (Xie et al., 2012) occurs through a salt bridge between a conserved Asp (D) or Glu (E) residue in the PHD finger and the K4 side chain (Fig. 3). These differences result from the action of the conserved residues of glycine (G) in PHD_BPTF_ and PHD_ING2_ or cysteine (C) in PHD_UHRF1_ and PHD1_KDM5B_. In PHD_BHC80_, this space is occupied by residue M502. In comparison, methylated H3K4me3 recognition was found to occur primarily through the interaction between the positively charged H3K4me3 and the conserved aromatic residues, such as W32 in PHD_BPTF_, W238 in PHD_ING2_ and W891 in PHD_TAF3_, as shown in Fig. 3. Other residues, including Y10 and Y17 in PHD_BPTF_, Y315 and M226 in PHD_ING2_, and W868 and M882 in PHD_ING2_, associate through hydrophobic interactions or aromatic Π-cation ion interactions to form the binding cage.

Thus, similar to PHD_BHC80_ and other PHD fingers (Fig. 3A), PHD1_KDM5B_ does not use the aromatic cage to specifically identify H3K4, which is present in structurally characterized methylated lysine PHD fingers, such as the PHD fingers in BPTF (i.e., PHD_BPTF_, PDB code: 2F6J), ING2 (i.e., PHD_ING2_, PDB code: 2G6Q) and TAF3 (i.e., PHD_TAF3_, PDB code: 2K17) (Fig. 3A) (Pena et al., 2006; Li et al., 2006). These PHD fingers adopt similar folds, engage the H3 peptide as an anti-parallel β-sheet on the surface and recognize the H3 N-amine and H3A1 side chain. H3R2 is buried in a pocket in PHD_BPTF_, PHD_ING2_, PHD_UHRF1_ (Hu et al., 2011; Wang et al., 2011; Lallous et al., 2011; Rajakumara et al., 2011; Xie et al., 2012) and PHD2_DPF3b_ (Zeng et al., 2010), bound in tiny surface grooves in PHD_AIRE_, PHD_TAF3_ and PHD1_KDM5B_, and not contacted in PHD_BHC80_.

The binding mode of PHD1_KDM5B_ to H3K4me0 is also distinguished from the caging of the di- and tri-methyl lysine by aromatic residues, as identified in the polycomb (Pc) and heterochromatin protein I (HP1) chromodomain (Fischle et al., 2003; Nielsen et al., 2002; Jacobs and Khorasanizadeh, 2002; Min et al., 2003; Tan et al., 2003). The methylation of lysine 9 in histone H3 is recognized by HP1; this methylation directs the binding of other proteins to control chromatin structure and gene expression. The structures of the complex between the *Drosophila* HP1 chromodomain and the histone H3 tail with a di- or tri-methylated K9 display histone tail inserts as a β strand, completing the β-sandwich architecture of the chromodomain. The methylated lysine is caged by the side chains of the aromatic residues Y21, W42 and F45, whereas adjacent residues form discerning contacts with one face of the chromodomain. The structure of the Pc chromodomain in complex with a H3 peptide bearing trimethylated K27 (Fischle et al., 2003) demonstrates that the methylated H3K27 is caged by four aromatic residues (i.e., Y26, W47, W50 and Y54) preceding the ARKS motif.

### Proposed biological function of PHD1_KDM5B_ in demethylation by KDM5B

The binding of the PHD1_KDM5B_ domain to the H3K4me0 peptide, which is a demethylation product of the full-length KDM5B, indicates that PHD1_KDM5B_ may function downstream of KDM5B demethylase activity. This observation is similar to that found for BHC80 in LSD1-mediated H3K4me2 demethylation and repression (Lan et al., 2007). The regulation of histone methylation is highly dynamic and involves the actions of both a methyltransferase and demethylase on identical target promoters. Therefore, we suggest that PHD1_KDM5B_ may be important in maintaining KDM5B at the target promoters and preventing the re-methylation of H3K4. The downstream effector of PHD1_KDM5B_ is therefore required for the KDM5B-induced demethylation of H3K4me2/3. This was confirmed by our binding assay results, which showed that the removal of PHD1_KDM5B_ resulted in decreased binding of KDM5B-N to the K4 unmethylated histone H3 peptide *in vitro* (Fig. 1C). Our findings indicate that PHD1_KDM5B_ is important for KDM5B association with its reaction product H3K4me0 after demethylation. Thus, PHD1_KDM5B_ operates both as a reader and a protector of unmethylated H3K4.

**Figure 5:**
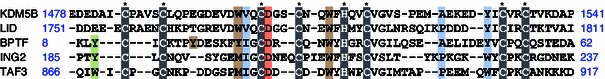
**Sequence alignment of PHD fingers (binding to H3K4me3/2) of KDM5B (i.e., PHD3**_**KDM5B**_**), Lid (i.e., PHD3**_**Lid**_**), BPTF, ING2 and TAF3**. The zinc-binding residues, H3A1-binding residues, H3R2-binding residues and H3K4me3/2-binding residues are highlighted in grey (and star on the top of KDM5B), blue, red and brown, respectively

Similar to PHD1_KDM5B_, PHD1_Lid_ has also been suggested to interact with H3K4me0 (Li et al., 2010). PHD1_Lid_ was proposed to bind to non-DNA elements, such as local chromatin environments, during H3K4me2/3 demethylation by Lid. The C-terminal PHD3 region of Lid (i.e., PHD3_Lid_) was observed to specifically bind to H3K4me2/3 through the interaction of aromatic residues in the PHD finger with the positively charged methylated H3K4. By aligning the amino acid sequences of PHD3_KDM5B_, PHD3_Lid_ and other PHD fingers that recognize the H3K4me2/3 peptide (Fig. 5), we found that the residues most likely interacting with the H3K4me2/3 site are highly conserved. Residues W1781 in PHD3_Lid_ and W1512 in PHD3_KDM5B_ are conserved corresponding to W32 in PHD_BPTF_, W238 in PHD_ING2_, and W891 in PHD_TAF3_. We thus suggested that PHD3_KDM5B_ might also specifically bind to methylated H3K4me2/3 and that the W1502 residue in PHD3_KDM5B_ may have a biological function similar to that of residues W1771 in PHD3_Lid_, Y17 in PHD_BPTF_, M226 in PHD_ING2_ and M882 in PHD_TAF3_. In mammalian cells, c-Myc prefers to bind to E-boxes located within a chromatin context that contain highly di- and tri-methylated nucleosomal histone H3K4 (Guccione et al., 2006). However, the mechanism through which Myc recognizes the chromatin landscape remains unclear. Here, we propose that KDM5B may utilize its H3K4me2/3-binding C-terminal PHD3 finger to tether Myc to its preferred chromatin context. This process may be enhanced by the interaction between PHD1_KDM5B_ and the unmethylated H3K4me0 N-terminal tail, thereby permitting the selection of biologically important E boxes. Further experiments are required to more precisely define the roles of KDM5B PHD fingers in cell growth.

In summary, we identified a specific interaction between PHD1_KDM5B_ and the unmodified H3K4 peptide. We further provided structural insights into the binding. The specific recognition of unmodified H3K4 by the PHD1 domain of KDM5B is important for the KDM5B histone demethylase activity in cells and for the transcriptional repression of tumor suppressor genes.

## Materials and methods

### PHD1_KDM5B_ preparation for crystallization and NMR experiments

The details of the preparation, purification, crystallization and determination of the PHD_KDM5B_ (residues 306–360 aa) X-ray structure were previously described (Guo et al., 2011). The modified pGEX-6p-1 vectors expressed proteins with the N-terminal GST tag, which is removable by cleavage with a 3C protease, enabling the use of non-tagged proteins in our studies. Only the X-ray structure of free PHD1_KDM5B_ was obtained.

A similar PHD_KDM5B_ construct was used for the sample required in the NMR experiments. ^13^C- and ^15^N-labeled PHD_KDM5B_ were prepared in M9 medium. Site-directed mutagenesis was performed using a QuikChange site-directed mutagenesis kit (Stratagene Inc. La Jolla, California, U.S.A). All of the DNA constructs were sequenced, and the molecular weights of the recombinant proteins were verified by mass spectrometry (MALDI).

### H3 peptide synthesis

To study the binding affinities *in vitro* or to construct different NMR samples of the complex, H3K4 peptides without modifications (with the ARTKQTARKS sequence or ARTKQTARKSTGGKAPRKQLA sequence) or with some modifications (H3R2me1, R2 monomethylation; H3R2me2s, R2 symmetric dimethylation; H3R2me2a, R2 asymmetric dimethylation; H3K4me1/2/3, K4 mono-, di-, and tri-methylation; H3K9me1/2/3, K9 mono-, di- and tri-methylation; H3 peptide A1G, R2A, T3V, K4A, Q5E, T6V mutants without any modification) were purchased from GL Biochem Ltd. (Shanghai, China), and their purity was confirmed by HPLC and mass spectrometry.

### GST pull-down and biotin pull down assays

For the biotin pull-down assay, biotin-labeled H3 peptides were used to pull down the PHD1_KDM5B_ protein in order to determine the binding affinities. Briefly, 0.5 μg of the peptide was mixed with the protein at an identical molar ratio, and the mixture was incubated with streptavidin beads at 4°C overnight. After washing five times, the beads were boiled in SDS loading buffer and separated on a SDS-PAGE gel.

For the GST pull-down assay, histone peptides (0.5 μg) were incubated with 2–5 μg of purified recombinant GST-PHD1_KDM5B_ for 2 h at 4°C in binding buffer (20 mmol/L Tris-HCl, pH 7.5, 150 mmol/L NaCl, 0.1% Triton X-100). The streptavidin beads (Upstate 16–126) were washed four times and stained with Coomassie blue.

### NMR and isothermal titration calorimetry (ITC) binding assay

The ^15^N-labelled PHD1_KDM5B_ and titrants (H3 peptide or its mutants) were mixed at a 1:6 molar ratio of PHD_KDM5B_:titrant in NMR buffer composed of 20 mmol/L Na_2_HPO_3_, 100 mmol/L NaCl, 0.01% NaN_3_, pH 7.4 and 10% D_2_O. The assignments of the cross peaks in the 2D ^1^H-^15^N HSQC spectrum were confirmed through NMR stepwise titration experiments using an increasing the molar ratio of PHD_KDM5B_:H3 as follows: 1:0.0, 1:0.6, 1:1.4, 1:2.0, 1:3.2, 1:6.2 and 1:7.2 (data not shown). The ^1^H-^15^N HSQC spectra were collected after each addition.

To investigate whether the PHD1_KDM5B_ finger and its mutants interact with unmodified or methylated H3K4 or H3K4mutants, the binding affinities of PHD1_KDM5B_ or its mutants to H3 peptides were studied. An ITC-200 microcalorimeter (GE Healthcare) was used with a buffer containing 20 mmol/L Tris, 150 mmol/L NaCl, and 1% Triton X-100, pH 7.5 at 25°C. The reference titration of small molecules in the buffer was subtracted from the experimental data, and the data were fitted using the Origin 7.0 (OriginLab Corporation) software. The results are summarized in Table S1.

### NMR spectroscopy and analysis

The NMR samples contained 1.5 mmol/L uniformly ^13^C/^15^N-labelled PHD_KDM5B_ and the unlabeled H3 peptide H3K4me0 in complex at a PHD_KDM5B_-to-H3-peptide molar ratio of 1:6 in NMR buffer (20 mmol/L Na_2_HPO_3_, 100 mmol/L NaCl, 0.01% NaN_3_, pH 7.4 and 10% D_2_O). All of the NMR experiments were performed at 20°C on a Varian Unity Inova 600 NMR spectrometer equipped with a triple resonances cryoprobe and pulsed field gradients. The standard suite of experiments for assigning the ^1^H, ^13^C and ^15^N backbone, determining the side-chain chemical shifts of PHD_KDM5B_ in complex with the H3 peptide and collecting the NOE-based distance restraints were measured (Bax and Grzesiek, 1993; Clore and Gronenborn, 1998), and these included 2D ^13^C-edited HSQC and ^15^N-edited HSQC; 3D HNCA, HNCO, HN(CO)CA, HNCACB, CBCA(CO)NH, ^15^N-resolved HSQC-TOCSY and HCCH-TOCSY in both aliphatic and aromatic regions; ^15^N-resolved HSQC-NOESY; ^13^C-resolved HSQC-NOESY for both aliphatic and aromatic resonances; and 2D hbcbcgcdceheA and hbcbcgcdhdA spectra for the correlation of Cβ and Hδ or Hε in the aromatic ring that is used for aromatic proton assignment (Yamazaki et al., 1993). The proton NMR signals of the bound H3 peptides were assigned by analyzing the 2D ^13^C-filtered, ^15^N-filtered and J-resolved NOESY and TOCSY spectra recorded for the ^13^C- and ^15^N-labeled protein with the unlabeled H3 peptide H3K4me0 and the 2D ^1^H-^1^H COSY, NOESY and TOCSY spectra recorded for the unlabeled free H3 peptides in the NMR buffer mentioned above, respectively. The intermolecular NOEs between the labeled protein and the unlabeled H3 peptides were obtained by analyzing the 3D ^13^C-F1 edited and ^13^C/^15^N-F3 filtered NOESY spectra. The spectra were processed with the NMRPipe program (Delaglio et al., 1995) and analyzed using Sparky 3 (http://www.cgl.ucsf.edu/home/sparky/).

### Determining the NMR structure

The calculations were performed using a standard simulated annealing protocol implemented in the XPLOR-2.19 program (NIH version)(Kuszewski and Clore, 2000). The inter-proton distance restraints derived from the NOE intensities were grouped into three distance ranges, namely 1.8–2.9 Å, 1.8–3.5 Å and 1.8–6.0 Å, which corresponds to strong, medium and weak NOEs, respectively. The dihedral angles phi and psi were derived from the backbone chemical shifts (HN, HA, CO and CA) using the program TALOS (Cornilescu et al., 1999). Slow-exchanging amide protons identified in the 2D ^15^N-^1^H HSQC spectra recorded after the H_2_O buffer was exchanged for a D_2_O buffer were used in the structure calculated with the NOE distance restraints to generate hydrogen bonds for the final structure calculation as previously described in the literature (Chakravarty et al., 2009). Constraints between the protein ligands and the zinc ion were added using a previously reported procedure (Neuhaus et al., 1992; Cao et al., 2003). A total of ten iterations were performed (50 structures in the initial eight iterations). In total, 100 structures were computed during the last two iterations, and the 20 conformers with the lowest energy were used to represent the 3D structures. The conformers of these two bundles (free PHD1_KDM5B_ and PHD1_KDM5B_ in complex with the H3K4me0 peptide) do not violate the following constraints: NOE > 0.3 Å and dihedral angle >3°. The entire structure statistics were evaluated with PROCHECK (Laskowski et al., 1993) and PROCHECK-NMR (Laskowski et al., 1996) and are summarized in Table 1. All of the structure figures were generated using the PyMOL (http://pymol.org/) and MOLMOL programs (Koradi et al., 1996).

### Immunofluorescence staining

The 293T cells were transfected with pcDNA3-KDM5B-Myc-His WT and mutants using Lipofectamine 2000. After 48 h, the cells were fixed with 4% paraformaldehyde for 10 min and permeabilized with 0.5% Triton in PBS for 15 min. After blocking, the cells were incubated with a primary antibody (Millipore, anti-H3K4me2, 1:200 dilution), and washed three times with PBS, and incubated with fluorescence-labeled secondary antibody for 1 h (Molecular Probes, Alexa Fluor 555 goat anti-rabbit, 1:250 dilution). After extensive rinsing with PBS, cover slips were mounted with an antifade reagent and DAPI (Molecular Probes) and examined on an Olympus IX51 microscope.

### Real-time qPCR assay

The pcDNA3-KDM5B-Myc-His WT and mutants were transfected into 293T cells with Lipofectamine 2000. Forty-eight hours after transfection, the total RNA was extracted using a standard protocol. Reverse transcription was conducted using the reverse transcriptase M-MLV from Promega. Real-time PCR was performed in triplicate using the SYBR Green PCR Mix (Promega) on an ABI 7500 sequence detection system (Applied Biosystems). Quantitative PCR reactions were performed under conditions that were standardized for each primer.

## Electronic supplementary material

Below is the link to the electronic supplementary material.

Supplementary material 1 (PDF 317 kb)

## Abbreviations

α-KG: α-ketoglutarate
JmjC: Jumonji C
LSD1: lysine-specific demethylase 1
WT: wild-type
