# Supplementary material for: The PHD1 finger of KDM5B recognizes unmodified H3K4 during the demethylation of histone H3K4me2/3 by KDM5B
Source: Protein Cell. 2014 Jun 22;5(11):837–50. doi: 10.1007/s13238-014-0078-4 (PMC4225485; doi:10.1007/s13238-014-0078-4)
Supplement: Supplementary file 1 — Supplementary material 1 (PDF 317 kb) [file 13238_2014_78_MOESM1_ESM.pdf]

The PHD1 finger of KDM5B recognizes unmodified H3K4 during demethylation of histone H3K4me2/3 by KDM5B

Yan Zhang[1 §], Huirong Yang[2 §], Xue Guo[2 §], Naiyan Rong[1], Yujiao Song[1], Youwei Xu[2], Wenxian Lan[1], Xu Zhang[3], Maili Liu[3], Yanhui Xu[2]*, Chunyang Cao[1]*

Supplementary materials and methods

^{1}H-^{15}N LR HSQC experiments of histidines in $PHD1_{KDM5B}$

To determine which nitrogen atom in the side-chain of His335 ligates to Zn^{2+} (Note, it is His335, not His344, which is conserved in JARID1 family member, thus His335 is more possible to ligate with zinc ion), we investigate the electronic properties of histidines in $PHD1_{KDM5B}$ in free state and in complex with unmodified H3K4me0 by performing two-dimensional ^{1}H-^{15}N LR HSQC experiments. The NMR sample contains ~0.5 mM $PHD1_{KDM5B}$ in NMR buffer (20mM Na_2HPO_3, 100mM NaCl, 0.01% NaN_3, pH 7.4 and 10% D_2O). The experiments were conducted at 20 ^{o}C on a Varian Unity Inova 600 spectrometer equipped with three channels and pulse-field gradient. This experiment was simply a conventional HSQC used for backbone amide correlations collected with an optimized two-bond $^{2}J_{NH}$ value of 22Hz in order to observe signals from the weak two-bond couplings in the histidine rings an suppress the signals from the one-bond J_{NH} amide couplings. The ^{15}N dimension was collected with 256 complex points, 120-ppm sweep width, 128 scans, and the ^{15}N carrier set at 205 ppm. The ^{1}H dimension was collected with 1024 complex points and 13.3-ppm sweep width centered at 4.82 ppm. The assignments were performed based on NOEs between the Hβ and aromatic proton Hδ. The final electrostatic properties of two histidines in $PHD1_{KDM5B}$ were identical, and the structure of the side-chain was drawn in the Figure S2.

Figure S1 Structural analysis of free $PHD1_{KDM5B}$. (A) Ribbon representation of the structure of free $PHD1_{KDM5B}$ determined by NMR (right) and X-ray crystallography

(left), respectively. (B) Comparison of free PHD1$_{KDM5B}$ structures obtained by NMR (red) and X-ray crystallography (blue), respectively, by superimposing the backbone C$_{\alpha}$atoms in secondary structural region. (C) Comparison of free PHD1$_{KDM5B}$ (red) and bound PHD1$_{KDM5B}$ (green) structures, by superimposing the backbone C$_{\alpha}$atoms in secondary structural region.

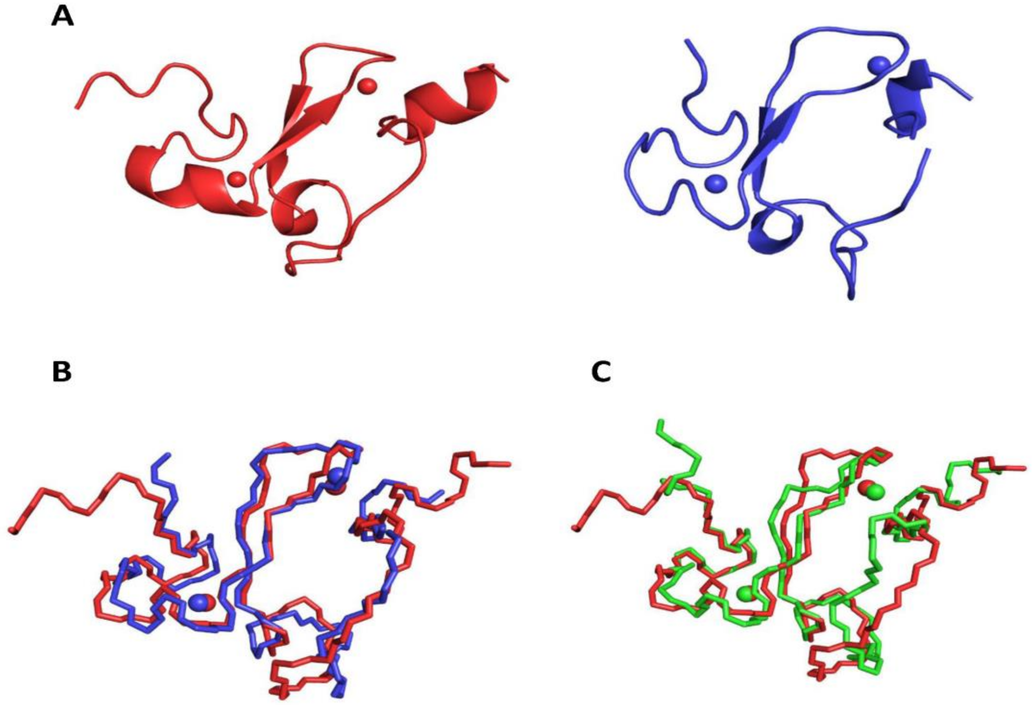

Supplemental Figure S2 ^{1}H-^{15}N LR HSQC experiments of histidines in PHD1$_{KDM5B}$. The final electronic property of histidine side-chain was drawn in the spectrum.

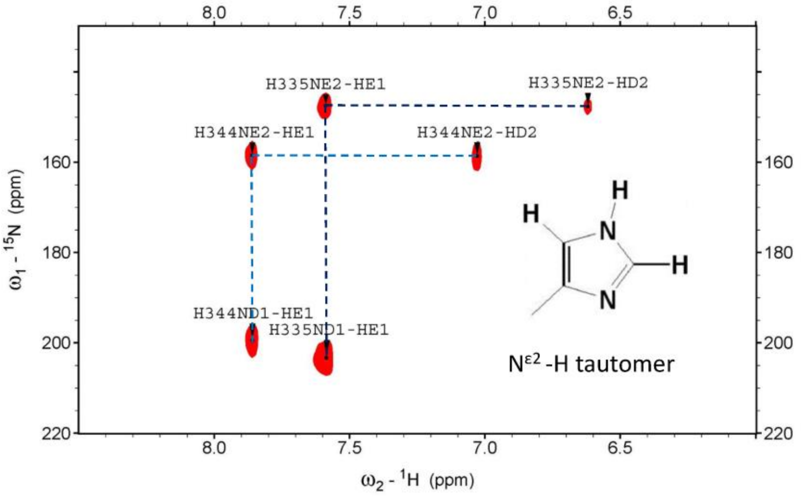

Table S1 Binding affinities for $PHD1_{KDM5B}$ or its mutants to H3 peptides (1-10aa).

KDM5B-PHD1 mutant	Peptide	K_D (μM)
KDM5B-PHD1	H3K4(1-10aa)	6.4 ± 0.6
KDM5B-PHD1-D308A	H3K4(1-10aa)	41.7 ± 2.2
KDM5B-PHD1-L309A	H3K4(1-10aa)	8.3 ± 0.5
KDM5B-PHD1-Y310F	H3K4(1-10aa)	4.3 ± 0.2
KDM5B-PHD1-Y310A	H3K4(1-10aa)	28.1 ± 1.3
KDM5B-PHD1-V311A	H3K4(1-10aa)	8.2 ± 0.3
KDM5B-PHD1-E321A	H3K4(1-10aa)	75.8 ± 5.7
KDM5B-PHD1-D322A	H3K4(1-10aa)	10.7 ± 0.5
KDM5B-PHD1-L324A	H3K4(1-10aa)	15.3 ± 0.7
KDM5B-PHD1-L325A	H3K4(1-10aa)	N. D.
KDM5B-PHD1-L326A	H3K4(1-10aa)	14.1 ± 0.8
KDM5B-PHD1-D328A	H3K4(1-10aa)	182.1 ± 21.9
KDM5B-PHD1-D332A	H3K4(1-10aa)	11.3 ± 0.5
KDM5B-PHD1-S333A	H3K4(1-10aa)	12.9 ± 0.5
KDM5B-PHD1-Y334A	H3K4(1-10aa)	12.6 ± 0.4
KDM5B-PHD1-D345A	H3K4(1-10aa)	6.7 ± 0.3
KDM5B-PHD1-W351A	H3K4(1-10aa)	N. D.
KDM5B-PHD1	H3K4 (1-10aa) A1G	304.9 ± 11.7
KDM5B-PHD1	H3K4 (1-10aa) R2A	370.4 ± 52.1
KDM5B-PHD1	H3K4 (1-10aa) R2E	N. D.
KDM5B-PHD1	H3K4 (1-10aa) T3V	57.1 ± 2.2
KDM5B-PHD1	H3K4 (1-10aa) K4A	15.9 ± 1.2
KDM5B-PHD1	H3K4 (1-10aa) K4E	N. D.
KDM5B-PHD1	H3K4 (1-10aa) Q5E	21.4 ± 1.7
KDM5B-PHD1	H3K4 (1-10aa) T6V	61.3 ± 1.6

N.D. means non-detectable
